# Drug Retention and Safety of Secukinumab in a Real-World Cohort of Ankylosing Spondylitis and Psoriatic Arthritis Patients

**DOI:** 10.3390/ijerph192315861

**Published:** 2022-11-29

**Authors:** Mateusz Moskal, Piotr Krawiec, Wojciech Zaręba, Izabella Świerczek, Jakub Ratusznik, Wiktor Raputa, Maciej Zieliński, Krzysztof Batko, Mikita Huk, Bogdan Batko

**Affiliations:** 1 Department of Rheumatology and Immunology, Faculty of Medicine and Health Sciences, Andrzej Frycz Modrzewski University, 30-705 Cracow, Poland; 2 Department of Research and Design, Medicine Economy Law Society (MELS) Foundation, 30-040 Cracow, Poland; 3 Department of Cardiology, J. Dietl Specialist Hospital, 31-121 Cracow, Poland

**Keywords:** secukinumab, real-world, psoriatic arthritis, ankylosing spondylitis, effectiveness, safety, drug retention, Central-Eastern European, electronic medical records

## Abstract

Real-life data that support effectiveness of secukinumab (SEC), an interleukin 17A inhibitor, in Poland are few. We aimed to evaluate SEC effectiveness based on drug retention and safety measures reported in electronic medical records (EMRs) of patients with psoriatic arthritis (PsA) and ankylosing spondylitis (AS) from two tertiary-care centers in the region of Lesser Poland. A total one-hundred eighty seven (127 PsA and 60 AS) first (*n* = 112), second (*n* = 59) and third-line SEC users were enrolled. The mean (SD) age of the sample was 45.7 (12.9), and 48% were male. All patients were classified with active and severe disease prior to initiation. Administrative delays for SEC users last a median 2 weeks. Median delay from symptom onset to diagnosis was 4 years (IQR 8), and differed by predominant disease subtype. The inefficacy rate was 10.7% and 18.6% for first and second-line users with median (IQR) drug maintenance estimated at 1.22 years (1.46) and 1.51 (1.38), respectively. First-year drug loss defined as drug switch due to inefficacy or adverse event was rare, with median estimates of 0.91 (95% CI; 0.85, 0.97) and 0.86 (95% CI; 0.77, 0.95) for first and second-line, respectively.

## 1. Introduction

Psoriatic arthritis (PsA) and ankylosing spondylitis (AS) are chronic inflammatory arthritides, which are characterized by systemic manifestations and progressive physical disability, all of which carry a significant added burden of disease [1,2,3,4].

Secukinumab is a monoclonal antibody that is fully humanized and acts to inhibit interleukin 17A (IL-17A). IL-17 is a crucial cytokine involved in the manifestations of spondyloarthropathy and psoriatic disease. IL-17, IL-22, IL-23 and TNF alpha intertwine in cytokine networks and are involved in initiating and maintaining skin and joint manifestations of these diseases [5,6,7,8,9]. While clinical trials have demonstrated that there is favorable efficacy and safety of secukinumab in both PsA and AS [10,11,12,13], data from the real-world are still emerging. These data are necessary to undertake decisions on both a expert (e.g., drafting recommendation) and regulatory (e.g., safety evaluation, local policy regulation, reimbursement, etc.) level. Therefore, studies performed from a regional perspective are crucial when considering the heterogeneity present across individual patients and healthcare (i.e., individual co-morbidities, disease severity, physician practices, local regulations, etc.).

In Central-Eastern European countries, as well as in low- to middle-income nations, the availability of novel antirheumatic drugs is often limited, while physician practices tend to be variable from nations with greater payer spending. An illustrative study has provided evidence for this claim, and has shown that biologic drug availability varies substantially across countries and healthcare [14]. Other reports, and our own epidemiologic studies have shown that studies in a given demographic are especially warranted, as the projected estimates extrapolated from other studies often do not fall in line with first-hand evidence [14,15,16,17,18,19]. Moreover, the importance of this study is justified by the limited therapeutic armamentarium currently available in Poland. Clarifying the efficacy and safety profile of novel agents may help regulators reduce the stringency of criteria for reimbursement, but also encourage physicians to chose novel agents over standard choices of therapy.

The aim of this study was to assess regulatory and diagnostic delays, as well as evaluate efficacy and safety of secukinumab in a real life cohort of patients with PsA and AS.

## 2. Materials and Methods

This was a retrospective cohort study, which aimed to assess delays in access to secukinumab, as well as real-life efficacy and safety based on information extracted from electronic medical records (EMRs) of two referral Rheumatology Clinics. Patients initiated secukinumab during the study recruitment period between January 2016 and January 2022. EMRs were analyzed to identify adult patients (>18 years of age) with AS and PsA. Patients were eligible for inclusion if they met the following criteria.

For PsA patients, fulfillment of ClASsification criteria for Psoriatic Arthritis (CASPAR) criteria was required [20]. Outcome measures treated as secondary efficacy endpoints included the provider assessed global acitvity (PhGA; 0–10 cm visual analogue scale(VAS)), pain (pVAS; measured on a 0–10 cm VAS), tender and swollen joint count (TJC/SJC, respectively), and the Bath Ankylosing Spondylitis Disease Activity Index (BASDAI) [21].

For AS patients, fulfillment of The Assessment of SpondyloArthritis International Society classification criteria (ASAS) [22], or modified New York criteria [23] criteria, as assessed by a specialist rheumatologist, was required. Outcome measures treated as secondary efficacy endpoints included PhGA, pVAS, TJC/SJC and the Bath Ankylosing Spondylitis Disease Acitivity Index (BASDAI) [21].

For AS patients, the inclusion criteria required the patient to have a documented fulfillment of all three criteria:

○ BASDAI ≥ 4 at least twice, with an interval of at least 4 weeks between assessments,
○ axial pain ≥ 4 on a VAS scale from 0 to 10 cm, assessed twice, with an interval of at least 4 weeks between assessments,
○ global assessment of disease (i.e., activity, severity, prognosis or work ability) score of over 5 on a scale from 0 to 10 cm, performed by two physicians, one of which was required to be an experienced specialist rheumatologist.

For PsA patients, the inclusion criteria necessitate documentation of active and severe disease, which is defined as:

If peripheral dominant, see criteria for AS, as reported above,

If axial dominant, evaluation by specialist rheumatologist at least twice with an interval of at least 4 weeks, without treatment adjustment in this timeframe. This point requires fulfillment of one of the following,

○ Disease activity score using 28-joint count (DAS28) greater than 5.1, or DAS greater than 3.1,
○ All of the following: SJC or inflammed tenditis documented in imaging-count of at least 5, TJC or tender tendons-count of at least 5, global assessment of disease activity by patient (PtGA) and physician (PhGA) over 3 in a 5 point Likert scale,
○ Global assessment of disease (activity, severity, prognosis or work ability) score of over 5 on a scale of 0 to 10 cm, as performed by two physicians, one of which is required to be an experienced specialist rheumatologist.

Data on co-morbidities was summarized according to the Charlson Comorbidity Index (CCI) due to its high inter-rater reliability and useful prognostic properties regarding long-term mortality [24].

The primary study endpoints were description of clinically significant delays in diagnosis and therapy access, as well as characterization of the inefficacy and safety profile of secukinumab as documented in EMRs.

For AS patients, inadequate response to treatment is defined as failure to achieve,

○ At 3 months (±2 weeks), reduction of BASDAI ≥ 50% for BASDAI.
○ At 6 months (±4 weeks), attaining low-disease activity defined as BASDAI < 3.

For PsA patients, inadequate response to treatment is dependent on the dominant disease subtype, but is based on maintained of active and severe disease (as defined above) despite treatment with a strategy in line with current EULAR/recommendations.

The study was conducted in compliance with the Declaration of Helsinki. Bioethics committee approval was not necessary due to retrospective character, which is confirmed by the Bioethics Committee decision (L.dz. OIL/KBL/35/2017). The report was written to include relevant points-of-interest based on the EULAR recommendations, which are an extension of the Strengthening the reporting of observational studies (STROBE) guidelines [25].

Analysis was performed using R (R Core Team, 2022. R Foundation for Statistical Computing, Vienna, Austria) and publicly available packages such as ggplot, tidyverse, rstatix and arsenal with subsidiaries. Continuous variables are summarized using the mean and standard deviation (SD), or median and interquartile range (IQR), as appropriate, while categorical variables are reported as counts and proportions (*n*, %). Comparison of categorical variables was performed using chi-square or Fisher’s exact test, while continuous variables are compared with variations of the linear model, or non-parametric equivalent, as appropriate.

## 3. Results

### 3.1. Administrative and Diagnostic Delays

Administrative delay was defined as time from application for treatment and the date of first drug dose admission. For first-line SEC users (*n* = 112), a median (IQR) delay of 14 days (13.2) was noted. Comparatively, a median (IQR) delay of 14 (17) days was recorded for first-line biologics (*p* = 0.121).

**Figure 1:**
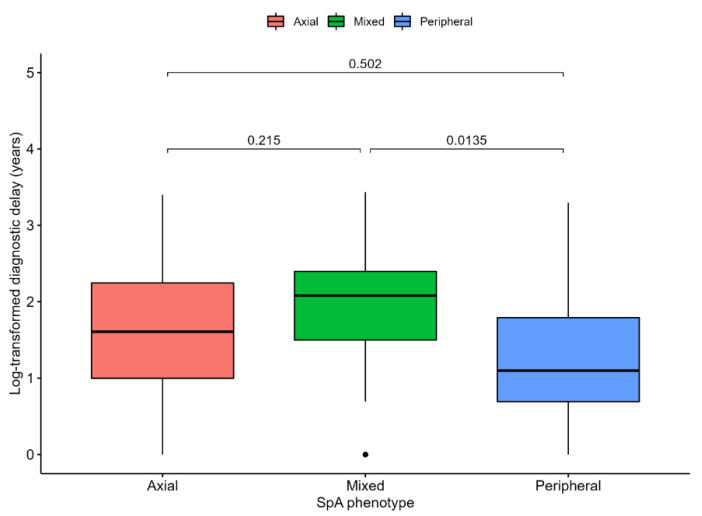
Diagnostic delay from symptom onset to disease diagnosis expressed in years and log-transformed (y axis is transformed to reflect a priori values) compared across spondylarthritis phenotype. ANOVA test *p* value = 0.016. *p*-values for comparison are shown and are based on Tukey’s post hoc tests.

The median delay from symptom onset to disease diagnosis was estimated at 4 years (IQR 8, range 30). In order to evaluate whether disease phenotype could translate into variable diagnostic delays, we performed a complete case analysis of the available data (*n* = 94). Log-transformed diagnostic delay was significantly associated with disease phenotype (*p* = 0.016), with Tukey’s post hoc tests indicating a significant difference between patients with mixed and peripheral disease, the latter of which experienced shorter delays (*p* = 0.014, details in Figure 1).

### 3.2. Demographic and Clinical Characteristics of Patients Treated with First-Line Secukinumab

An overall summary of clinical variables is provided in Table 1 and a comparison between patients with PsA and AS was performed. In this sample, patients with PsA were significantly older and more commonly female. Acute-phase reactants were numerically higher in AS patients, but overall, a modest elevation can be observed for the average patient. Pain intensity was relatively high and consistent across groups. Manifestations of spondyloarthropathy are different across diseases, as can be expected from different underlying pathogenetic pathways. Dactylitis and skin psoriasis were significantly more common in PsA cases. In turn, HLA-B27 positivity and uveitis were much more common in AS.

A comparison of baseline and 6-month values indicates improvements in pain, axial symptoms and acute phase reactants of secukinumab users. Specifically, there was a significant difference between T0 and T6 for log-transformed CRP (*n* = 87, *p* = 0.013), ESR (*n* = 87, *p* = 0.013), pain (*n* = 104, *p* < 0.001) and BASDAI (*n* = 104, *p* < 0.001). Analysis of composite indices of disease activity was not performed due to infrequent reporting.

Higher starting dose was more commonly preferred in PsA, while dose change occurred in approximately one third of patients, with similar rates in both diseases. Regarding adjunct treatment, glucocorticoids were maintained more often in PsA, as opposed to AS. The rate of NSAID treatment was low, at approximately 10% in both groups.

### 3.3. Secukinumab Treatment Safety and Drug Retention as a Measure of Effectiveness

One hundred and twelve patients received SEC as first-line treatment, while fifty-nine subjects (~30%) received it in second, and sixteen (~10%) in third-line. Median (IQR) duration of treatment maintenance was 1.22 years (1.46) for first, 1.51 (1.38) years for second and 1.86 years (1.94) for third-line.

**Figure 2:**
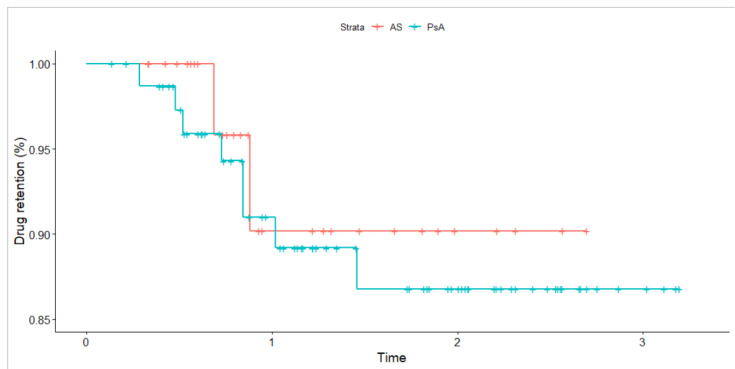
Crude Kaplan–Meier survival curve for drug retention compared across disease types for patients receiving secukinumab in first-line. Time is expressed in years. The *p*-value based on the log-rank test is 0.68. Tick marks indicate censored events.

The median drug survival estimate at six months was 0.98 (95% CI; 0.96, 1.00) and 0.91 (95% CI; 0.85, 0.97) for year one in first-line. There was no significant difference in drug retention as compared using crude Kaplan–Meier survival curves and the corresponding log-rank test (see Figure 2). The vast majority (*n* = 100/112, 89.3%) of patients in the first-line SEC group did not have a record for inefficacy. Of these patients, 89 (89%) individuals already maintained the drug over 6 months. Only 2 (3%) cases of significant adverse events (SAE) (both cases of an anaphylaxis-like reaction) that necessitated drug change were recorded in first-line subjects. Table A1 provides a detailed description of SAE characteristics. The incidence rate for any SAE was estimated at 12.12 (95% CI 1.47, 43.8) per 1000-person years.

**Figure 3:**
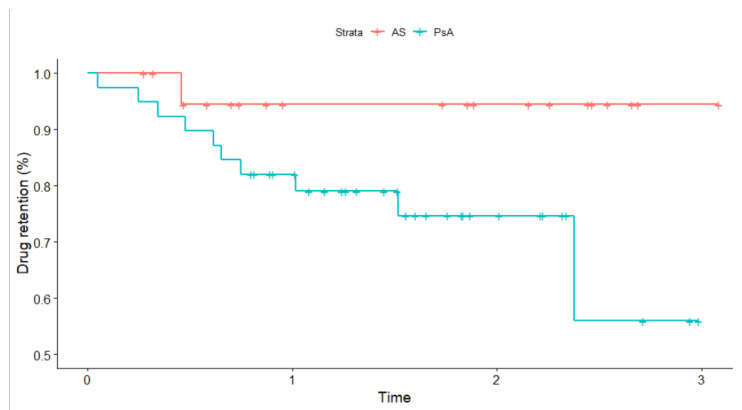
Crude Kaplan–Meier survival curve for drug retention compared across disease types for patients receiving secukinumab in second-line. Time is expressed in years. The *p*-value based on the log-rank test is 0.07. Tick marks indicate censored events.

The median drug survival estimate at six months was 0.91 (95% CI; 0.84, 0.99) and 0.86 (95% CI; 0.77, 0.95) for year one in second-line (see Figure 3). The vast majority (*n* = 48/59, 81.4%) of patients in second-line SEC did not have a record for inefficacy. Of these patients, 45 (94%) individuals already maintained the drug over 6 months. In second-line, three SAE (5%) were reported (two cases were serious and recurrent fungal infections, one was gastrointestinal). The incidence rate for any SAE was estimated at 34.25 (95% CI 7.06, 100.08) per 1000-person years.

## 4. Discussion

The current report is one of the first to provide evidence regarding real-world efficacy and safety of SEC as a first- and second-line agent in a clinical cohort of Polish PsA and AS patients, of which the vast majority have initiated and maintained the drug for over 6 months. The salient finding of this study is an indication of a low rate of treatment failure, defined as lack of inefficacy or adverse events (i.e., necessitating drug change for secukinumab), in general. Comparable drug retention was observed for SEC as a first-line choice in both AS and PsA, while conversely, potentially less favorable results are achieved in PsA with SEC as a second-line agent. However, the inefficacy rate in PsA second-line users is still relatively low, which supports a favorable profile of SEC. It should be noted that the modest sample size precludes any definite conclusions and is inherently tied to a degree of uncertainty that warrants further replication of these findings.

Inhibition of the proinflammatory IL-17A cytokine is an effective therapeutic modality in PsA and AS. There is an accrual of evidence from clinical trials [10,11,12,13], but real-life data are still few [26,27]. Recent pan-registry analyses have shown 6- and 12-month retention rates are high, while overall effectiveness is comparable to data for TNF inihibiting agents, which is encouraging [28]. However, while registry-linkage is undeniably a robust source of information, it often does not include particular demographics, for which it is more difficult to gather data, and at the same time, such data are needed to undertake regulatory and reimbursement decisions. The present study identifies a satisfactory retention rate for patients with PsA and AS, as compared with other real life cohorts [27,29]. Our results fall in line with recent studies that indicate SEC can be used in both biologic naïve and experienced patients [28].

Safety analyses from PsA and AS have been systematically analyzed based on pooled data from SEC trials [30]. The upper 95% CI for serious and fungal infections ranged between 1.6–2.8 and 1.2–2.5 per 1000 person years in PsA and AS, respectively. This is comparable to what we observed for second-line SEC users regarding infection incidence, since this was the only type of SAE observed in second-line. Conversely, trial safety data show that the cumulative rate of hypersensitivity reactions can be estimated at 0.24 per 1000 person years [30], which is considerably lower than our own observations, but considering the small sample the degree of uncertainty is high, which is reflected in the confidence intervals around the estimate itself. It should be emphasized that the safety events reported in the current study are adverse effects that warranted reporting in medical records and are unlikely to be representative of all side-effects experienced by patients. Due to patient follow-up at sequential visits, patient recall and attribution bias needs to be considered. From a provider perspective, reporting bias may be present in that mild to moderate side-effects that do not necessitate drug switch may not be reported consistently. These factors need to be taken into account when interpreting the results of the present study.

Recent data from the US show that secukinumab is effective in a real-world setting of PsA patients who maintain the drug for at least 6 months, with at least one third achieving minimal disease activity. It should be noted that most (>80%) patients had prior biologic treatment (60% at least third-line) [31]. As such, a comparison with the Polish demographic remains difficult. The availability of biologics is low in Poland, while the stringency of drug reimbursement is high [16]. The patients from the present study are likely to have difficult to control disease by conventional measures, which is likely incomparable to inadequate control from other nations. It should be noted that the likelihood that providers would maintain patients on secukinumab when it was in fact ineffective (i.e., due to a lack of other reliable treatment options) is low, as all TNF inhibitors are reimbursed and tofacitinib was also available throughout the period of this study. To illustrate the differences by healthcare and welfare, we can consider an illustrative example of achieving a state of difficult-to-treat rheumatoid arthritis, as per the recently published EULAR guidelines [32]. Considering biologic experienced patients consist ~3% of the Polish RA population [16], and that only a proportion will experience inefficacy to at least two agents, this population is likely significantly different than from that of other nations where up to one third of patients could be categorized as such [33,34]. Therefore, when comparing across studies it is necessary to understand the regional context of inadequate disease control through consideration of real life b-/tsDMARD availability. These cross-national differences are reflected by the fact that the majority of patients in the present study are first-line users, as compared to other real-world cohorts, where the majority are biologic experienced [26,31,35]. Further, in US cohorts the majority of patients are third-line biologic users, with little data available for first-line cases [31].

In order to understand the underlying patient demographic, we analyzed administrative and diagnostic delays. Prior studies have shown that more than 6 months of diagnostic delay is tied to poor prognosis regarding radiographic and functional outcomes in PsA [36]. In other European nations, diagnostic delays in PsA were similar to our results with a mean (SD) estimate of 4.01 (1.42) years for Spain [37], 3.41 (4.75) for Denmark [38] or median 1 year (IQR 0.5–2) for Ireland [36]. A study in the United Kingdom reported a symptom duration of over one year prior to diagnosis in close to one third of patients [39]. In US cohorts, median time from symptoms to diagnosis was 2.5 years (IQR 0.5–7.3) [40]. Similarly, in AS the average diagnostic delay estimates ranges over years with cohorts reporting mean (SD) estimates of 6.05 (5.08) years [41], or even 7.88 (7.17) [42]. Systematic review in axial spondylarthritis have shown that delays have considerable variability, with the majority of studies reporting median delays between 2 and 6 years [43].

The results of the current report may not reflect all patients with PsA and AS in the Central-Eastern European setting, or even for Poland, as data were gathered from to reference centers, in which the patient and provider demographic is also likely to be different. The present study is also limited by lack of a comparator cohort for other biologics, which makes it difficult to benchmark measures of effectiveness and safety. It should be noted that there is a steady increase in the recorded prevalence of PsA in Poland [44,45], while the current treatment armamentarium remains limited. More data are necessary to support the decisions of providers and policy makers to enable reimbursement of novel and effective therapies. The sample size of the present study was modest, particularly for third-line SEC users, which is of importance from a comparative perspective. It is necessary to treat this report as suggestive of secukinumab efficacy, and encouraging to gather further prospective and/or nationwide evidence.

## 5. Conclusions

This report provides real life evidence of a low rate of treatment failure, defined as inefficacy or adverse event necessitating drug switch, for secukinumab in PsA and AS patients. The vast majority of patients analyzed at present maintained secukinumab over six months, and the estimate for drug retention in both first and second-line is high, regardless of underlying disease type. Furthermore, regulatory delays from drug admission to availability may last up to a few weeks. Diagnostic delays recorded in the current patient sample are significant with a median of 4 years, which likely illustrates difficulties in access to a specialist, diagnostic problems and/or clinical inertia.

## Figures and Tables

**Table 1:** Baseline comparison of patients with ankylosing spondylitis and psoriatic arthritis treated with secukinumab including both first- and second-line cases.

	AS (*n* = 60)	PsA (*n* = 127)	Total (*n* = 187)	*p* Value
Age (years)	40.5 (10.8)	48.2 (13.1)	45.7 (12.9)	<0.001
Sex (Male)	41 (68.3%)	49 (38.6%)	90 (48.1%)	<0.001
CRP at first SEC dose (baseline) *	17.65 (23.15)	8.52 (13.11)	11.78 (17.78)	0.281
CRP at 6 months following first SEC dose (T6) *	6.50 (10.45)	4.35 (3.58)	5.12 (6.88)	0.966
ESR at first SEC dose (baseline) *	14.12 (16.71)	13.09 (12.31)	13.47 (14.00)	0.693
ESR at 6 months following first SEC dose (T6) *	8.94 (6.49)	10.44 (9.61)	9.89 (8.58)	0.906
Pain at first SEC dose (baseline) **	66.77 (22.17)	67.05 (17.32)	66.93 (19.32)	0.942
Pain at 6 months following first SEC dose (T6) **	21.29 (15.57)	17.69 (14.64)	19.15 (15.05)	0.253
BASDAI *** at first SEC dose (baseline)	6.67 (1.73)	6.74 (1.38)	6.71 (1.53)	0.806
BASDAI *** at 6 months following first SEC dose (T6)	2.08 (1.36)	1.99 (1.32)	2.03 (1.33)	0.721
Enthesitis (Present)	7 (11.9%)	21 (16.8%)	28 (15.2%)	0.384
Dactylitis (Present)	4 (6.8%)	44 (35.2%)	48 (26.1%)	<0.001
Psoriasis (Present)	6 (10.2%)	90 (72.0%)	96 (52.2%)	<0.001
HLAB27 (Present)	38 (76.0%)	29 (35.4%)	67 (50.8%)	<0.001
IBD (Present)	2 (3.4%)	3 (2.5%)	5 (2.8%)	0.727
Uveitis (Present)	7 (11.9%)	3 (2.5%)	10 (5.6%)	0.010
CCI Index				0.102
None	52 (86.7%)	92 (73.6%)	144 (77.8%)	
One	7 (11.7%)	24 (19.2%)	31 (16.8%)	
Two or more	1 (1.7%)	9 (7.2%)	10 (5.4%)	
NSAID at present (Yes)	8 (13.3%)	17 (13.9%)	25 (13.7%)	0.912
GC at present (Yes)	6 (10.0%)	27 (22.1%)	33 (18.1%)	0.046
csDMARD at present (Yes)	7 (11.9%)	37 (30.3%)	44 (24.3%)	0.007
SEC line (First)	28 (46.7%)	47 (37.0%)	75 (40.1%)	0.208
SEC dose (Higher)	6 (10.5%)	57 (48.7%)	63 (36.2%)	<0.001
SEC dose change (Yes)	20 (35.7%)	36 (31.9%)	56 (33.1%)	0.616

Abbreviations: Charlson Comorbidity Index, CCI; conventional synthetic disease modifying agent, csDMARD; C-reactive protein, CRP; erythrocyte sedimentation rate, ESR; glucocorticoid, GC; human leukocyte antigen, HLA; inflammatory bowel disease, IBD; nonsteroid anti-inflammatory drugs, NSAID; secukinumab, SEC * Data was available only for 32 and 55 AS and PsA patients, respectively. ** Data available for 42 and 62 patients with AS and PsA, respectively. *** Data available for 44 and 60 patients, respectively. Group comparison is performed with *t* test and chi-square for continuous and categorical variables, unless otherwise indicated. Some variables were log-transformed due to right-skewed distributions.

## Data Availability

Data is available upon request.

## Appendix A

**Table A1:** Description and characteristics of significant adverse events necessitating drug switch for secukinumab users.

Rheumatic Disease	Time to Record Report	Adverse Effect	Drug Line
PsA	79 days	Ungual edema, erythema, dyspnea shortly following drug admission	First
PsA	50 days	An episode of dyspnea, blood pressure spike and syncope reported between second and third drug admission	First
PsA	91 days	Recurrent fungal infection of the oral cavity necessitating oral antifungal treatment	Second
PsA	126 days	Suspected gastrointestinal infection with severe stomach pain, diarrhea and hematochezia	Second
PsA	18 days	Massive fungal infection of the oral cavity, aphthous ulcers, peripheral extremity exanthem with ulceration	Second
AS	196 days	Cutaneous exanthem and stomach pain associated with drug admission	Third

Abbreviations: AS, ankylosing spondylitis; PsA, psoriatic arthritis.
